# Double-Blind, Randomized, Controlled Pilot Trial to Specify Collateral Effect and Safety of Perioperative Dexmedetomidine in Patients Undergoing Open Heart Surgery

**DOI:** 10.5812/aapm-157117

**Published:** 2025-02-22

**Authors:** Bassim Mohammed Jabbar Hatemi, Ayesheh Enayati, Somayeh Ghorbani, Fatemeh Tahmasebi, Hadi Abo Aljadayel, Ali Jabbari, Ali Movafegh

**Affiliations:** 1 Ischemic Disorders Research Center, International Campus, Golestan University of Medical Sciences, Gorgan, Iran; 2 Department of Anesthesia Techniques, College of Health and Medical Techniques, Al-Mustaqbal University, Babylon, Hillah, Iraq; 3 Ischemic Disorders Research Center, Golestan University of Medical Sciences, Gorgan, Iran; 4 Cancer Research Center, Golestan University of Medical Sciences, Gorgan, Iran; 5 Department of Anesthesiology and Critical Care Medicine, Ischemic Disorder Research Center, Golestan University of Medical Sciences, Gorgan, Iran; 6 Department of Anesthesiology, School of Medicine, Tehran University of Medical Sciences, Tehran, Iran

**Keywords:** Dexmedetomidine, Collateral Effect, Open Heart Surgery, Hemodynamic Parameters, Circulation

## Abstract

**Background:**

This study aimed to assess the collateral effects and safety of dexmedetomidine (Dex) during and one day after surgery in Iranian patients undergoing open heart surgery, to expand the clinical applications of Dex in Iran.

**Methods:**

This pilot study was conducted in Gorgan, Golestan province, Iran, in 2024. Both male and female participants undergoing open heart surgery were randomly assigned to either the Dex group (n = 10), receiving 0.5 µg/kg/h along with standard anesthesia management, or the control group (n = 10). The primary outcome was the percentage of patients experiencing adverse events. Secondary outcomes included the stability of hemodynamic and respiratory parameters, the occurrence of arrhythmias, and biological changes assessed during and 24 hours after surgery.

**Results:**

Out of 45 participants, 20 were enrolled and analyzed. The comparison between groups showed that observed adverse effects were higher in the control group (4 patients) compared to the Dex group (1 patient), with common events being hypotension, bradycardia, and tachycardia. Biological markers, such as lactate and blood sugar (BS), increased in both groups, with the control group showing a greater increase in both lactate and BS levels (P = 0.012 and P = 0.009, respectively) compared to the Dex group (P = 0.017 and P = 0.093, respectively). Additionally, there were no significant differences in hemodynamic and respiratory markers between the groups (P > 0.05); however, Dex improved and preserved hemodynamic and respiratory stability more effectively.

**Conclusions:**

The addition of Dex to the anesthesia protocol was safe and without adverse events, showing a promising role in improving cardiac function in patients undergoing open heart surgery.

## 1. Background

Coronary artery bypass grafting (CABG) is an effective method for treating coronary artery stenosis (1). Cardiac surgeries, such as valve replacement and CABG, are associated with a high incidence of cardiovascular and other complications during the perioperative period, leading to increased mortality and prolonged hospital stays. To prevent these adverse events, safe and comprehensive perioperative management is required (1). Myocardial damage may occur due to the standard surgical method, which involves performing cardiopulmonary bypass (CPB) during cardiac arrest (2). Additionally, hemodynamic changes during surgery can lead to myocardial ischemia (3). Perioperative myocardial ischemia, which develops in the presence of hemodynamic disturbances, is more commonly associated with tachycardia rather than hypotension or hypertension (3). Furthermore, cardiac dysfunction or surgery increases inflammatory mediators and reactive oxygen species in the heart, likely contributing to impaired cardiac pump function (4). Employing beneficial anesthesia and operative strategies to protect the heart during open heart surgery by attenuating reperfusion injury and systemic inflammatory response is essential to reduce morbidity (4). Although many anesthetics have cardioprotective effects, the variety of proposed protective mechanisms—such as attenuation of Ca^2+^ overload, anti-inflammatory and antioxidant effects, and pre/postconditioning-like protection — may have contributed to the slow adoption of anesthetics as cardioprotective agents in open heart surgery (4).

Dexmedetomidine (Dex) is an imidazole compound and a selective α2-adrenoceptor agonist. It is a potent agent widely used for sedation, anesthesia, and as an antioxidant, anti-inflammatory, and sympatholytic in surgeries (5, 6). Additionally, Dex attenuates the hemodynamic stress response to intubation, surgical stress, and extubation through its sympatholytic effect (5, 6). Its mechanism of action is unique and differs from those of other sedative agents (7). Activation of the receptors in the brain and spinal cord inhibits neuronal firing, resulting in hypotension, bradycardia, sedation, and analgesia, as well as reducing lactate levels and blood sugar (BS) (7, 8). Dexmedetomidine does not have a direct effect on myocardial contractility. It also exhibits a vasodilatory effect by activating alpha-2 adrenoceptors in endothelial cells (9). By decreasing the plasma level of norepinephrine, Dex provides perioperative cardiac protection by lowering blood pressure (BP) and heart rate (HR), thereby improving the oxygen supply-demand balance of cardiac muscle and decreasing serum troponin levels (10). A biphasic BP response is observed following rapid administration or at a high dose (> 1000 µg/kg). The Dex causes a biphasic BP response, with α-2A adrenergic receptors mediating the subsequent hypotension and α-2B adrenergic receptors causing the initial brief phase of hypertension (11). This direct action on the smooth muscle of the peripheral vessels typically lasts up to ten minutes (11). The Dex is described as an ideal medication in the perioperative period for managing wedge pressures (12, 13).

The incidence of hypotension and bradycardia may be related to the administration of a large intravenous "loading" dose of Dex (14). Omitting the loading dose or administering no more than 0.4 µg/kg of Dex can reduce the incidence or severity of hypotension. Administering the loading dose over 20 minutes also minimizes transient hypertension (14, 15). Conversely, Dex has been shown to reduce perioperative oxygen consumption and blunt the sympathetic response to surgery, potentially improving cardiac outcomes (15, 16). The effects of Dex on the cardiovascular system are dose-dependent. A well-known adverse effect of Dex at lower infusion rates is a reduction in HR and BP due to systemic effects (14). Higher doses primarily have peripheral vasoconstrictive effects, which increase BP and vascular resistance in the systemic circulation while also enhancing the effect of a slowing HR; therefore, caution is advised in patients with severe heart block or vasoconstriction (17). Consequently, a typical adverse effect of administering Dex is a reduction in HR. Its bradycardic impact may be due to the inhibition of sodium channels and acetylcholinesterase receptor channels, in addition to its central α-2 blocking effects (17). The Dex causes a dose-dependent decrease in vasoconstriction and shivering thresholds but does not affect sweating. α-2 adrenergic agonists reduce thermosensitivity at spinal and supraspinal locations by reducing neuronal conductance (11).

The Dex is known to modulate cardiac electrophysiology by limiting the function of the sinus node and atrioventricular node, as well as influencing myocardial repolarization (18). Previous studies have demonstrated that Dex can reduce postoperative tachyarrhythmia. Results from a meta-analysis showed that perioperative Dex administration can lower the risk of postoperative ventricular tachycardia and delirium in patients undergoing cardiac surgery, although it may also increase the risk of bradycardia (19, 20). Estimates indicate a lower risk of atrial fibrillation, a shorter length of hospital and intensive care unit (ICU) stay, and a higher risk of hypotension with the use of Dex (19-22). Moreover, another meta-analysis demonstrated that treatment with Dex was associated with an increased risk of bradycardia while lowering HR, systolic BP, and the incidence of tachycardia and arrhythmias (19-22). Additionally, the study suggests that Dex is a useful medication for cardioprotection in patients undergoing cardiac surgery, in both adult and pediatric populations (23).

## 2. Objectives

Due to the several effective roles of Dex during and after surgery, we designed a pilot randomized controlled study to evaluate the collateral effects of Dex during induction, maintenance, and postoperatively in Iranian patients undergoing open heart surgery, aiming to expand the clinical applications of Dex in Iran.

## 3. Methods

### 3.1. Study Design

We conducted a pilot double-blind randomized controlled trial on open heart surgery patients from June 2024 to August 2024. This single-center study was performed on patients admitted to the Sayyad Medical and Educational Center, Golestan University of Medical Sciences, Iran.

### 3.2. Study Population

A total of 20 patients undergoing open heart surgery (on-pump coronary artery bypass graft surgery, valvular repair surgery, and valvular replacement surgery), both male and female, aged 18 - 65 years, were included in this trial. Patients with serious coagulopathy or bleeding, inflammatory diseases, a history of arrhythmia, severe complications during surgery, seizure disorders, rheumatic disorders, chronic renal failure, concurrent surgeries, psychiatric disorders, alcoholism, and those requiring more than one valve repair were excluded.

### 3.3. Ethics

The study was approved by the Ethics Committee of Golestan University of Medical Sciences (IR.GOUMS.REC.1403.128) and registered at the Iranian Registry of Clinical Trials (IRCT20240624062233N1). All subjects provided written informed consent prior to receiving anesthesia.

### 3.4. Sample Size

To investigate the side effects of Dex in cardiac surgery, patients were divided into two groups, each with a sample size of 10 patients. In a clinical trial conducted in Japan to assess the efficacy and safety of Dex for procedural sedation in patients receiving local anesthesia outside the ICU, bradycardia was the most significant adverse event, occurring more frequently in the Dex group than in the control group (23). In the study by Zhai et al. (24), changes in HR were examined immediately after sternal closure, and at 4, 12, and 24 hours post-surgery. Considering the mean (standard deviation) HR immediately after sternal closure of 59.83 (3.66) beats per minute in the Dex group and 68.06 (4.49) beats per minute in the placebo group, a sample size of 6 patients per group was required to detect between-group differences at a two-sided significance level of 5% with 80% power, based on the *t*-test. Using G*Power software, the sample size for HR changes 4 hours after surgery was also calculated, and the maximum was selected as the sample size for this study. Therefore, to increase the study's power and account for potential exclusions and dropouts, we increased the sample size to 20 patients (10 patients in each group).

### 3.5. Anesthesia Management

In the Dex group (intervention), patients received a 0.5 µg/kg/h infusion of Dex, along with midazolam (0.1- 0.2 mg/kg/h) and sufentanil (3 - 5 µg/kg/h) infusions. The control group received isoflurane 0.4% as an inhalational agent, in addition to midazolam (0.1 - 0.2 mg/kg/h) and sufentanil (3 - 5 µg/kg/h) infusions, adjusted according to patient condition. Both groups received etomidate 0.3 mg/kg, midazolam (0.1 - 0.15 mg/kg) intravenously, sufentanil 1 µg/kg, and cisatracurium (0.15 - 0.2 mg/kg) for induction of anesthesia.

### 3.6. Randomization and Blinding

**Figure 1.**
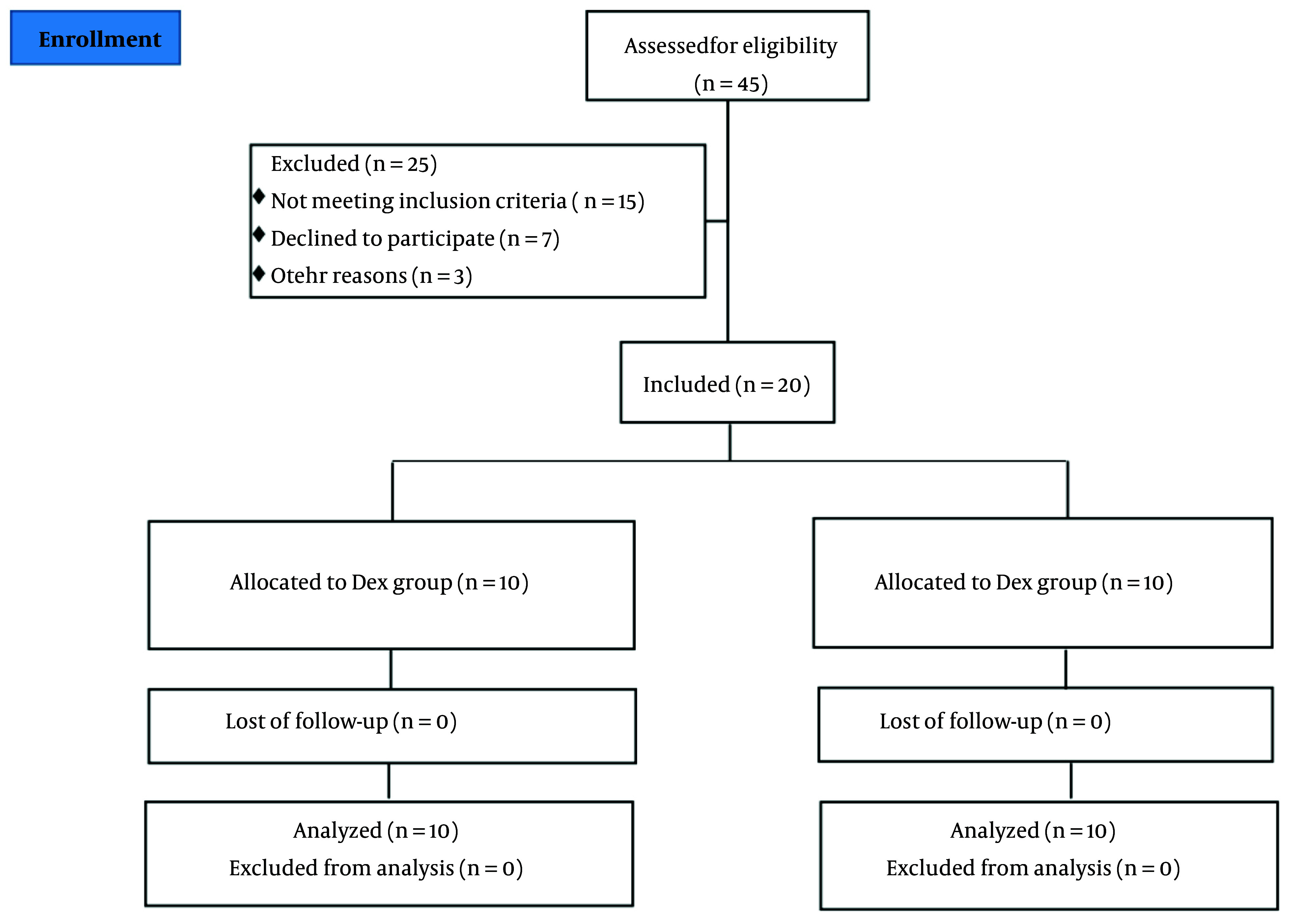
CONSORT flow chart

Eligible patients were divided into two groups using a random block design. An online service tool (sealedenvelope.com) was used to create the blocked randomization list. To prevent guessing which treatment each patient received, the block size was set to 4, a multiple of the number of treatments. Treatments were assigned to patients according to the generated list. To conceal the randomization sequence until the start of the study, consecutively numbered opaque envelopes containing cards with the treatment type were used. Patients and anesthesiology technicians, who measured the primary and secondary outcomes of the study, were blinded to the type of anesthetic each patient received. However, the anesthesiologist was aware of the treatment received so that, in case of a problem, they could break the randomization code and take necessary supportive measures. Figure 1 shows the study flowchart.

### 3.7. Outcomes

The primary endpoint was the percentage of patients who exhibited adverse effects during Dex administration and post-operation. The secondary endpoints included vital signs, such as hemodynamic parameters (HR, BP, and mean arterial pressure), respiratory characteristics [blood oxygen saturation (SpO_2_) and partial pressure of carbon dioxide (PaCO_2_)], arrhythmia incidence, and laboratory information (lactate, BS). These were measured before, during, and after the induction of the drug and in the postoperative phase. Additionally, safety assessments and the incidence of adverse effects, including hemodynamic and respiratory parameters and biological markers during and after the operation, were defined as shown in Appendix 1 in Supplementary File, based on the study by Inagaki et al. (23).

### 3.8. Statistical Analysis

After data collection, analyses were conducted using IBM SPSS Statistics 23 software. The normality of quantitative data was assessed by drawing histograms and Q-Q plots. Quantitative variables with a normal distribution were described by mean ± standard deviation (SD), while categorical variables were described by median (first and third quartiles) and frequency (percentage). Fisher's exact test and chi-square test were used to compare categorical variables such as gender, number of diabetic and hypertensive patients, and adverse treatment effects between the two trial arms. A *t*-test was used to compare normally distributed continuous variables between the two groups. Skewed quantitative variables were compared using a nonparametric Mann-Whitney test. In paired cases, a Wilcoxon signed-rank test was used to compare variable levels before and after pumping and at other paired times within each group. Since all patients received treatment according to the randomization list and there was no change in the treatment assigned during surgery, an intention-to-treat (ITT) analysis was used. The outcomes of the experimental and control groups were compared regarding differences in variable levels from pre-pump-on to post-pump-off, using the Mann-Whitney test for skewed data, and described with median (first and third quartiles). The Friedman test was used to compare repeated measures at different times within groups. A P-value of < 0.05 was considered statistically significant. The two-sided tests were considered because the superiority of the experimental and control groups was questionable.

## 4. Results

### 4.1. Baseline Characteristics

Between June 2024 and August 2024, a total of 45 elderly patients who underwent cardiac surgery were assessed, and 20 patients were included in the study. Twenty-five individuals were excluded from the trial for various reasons. Consequently, 20 patients were randomly assigned to the study groups and underwent full analysis [control group (n = 10) and Dex group (n = 10)]. At baseline, demographic and clinical characteristics were compared between the groups and were found to be similar (Table 1 and Table 2). 

**Table 1.** Patient’s Demographic Characteristics ^a^

Characteristics	Groups		P-Value
	Control (n = 10)	Dex (n = 10)		
**Age (y)**	58.88 ± 8.17	58.90 ± 8.02	0.995
**Male/female**	4/6	6/4	0.371
**Original surgical procedures**			
CABG	6 (60)	8 (80)	
CABG + AVR	0	0	
TVR + MVR	1 (10)	0	
CABG + ASD	1 (10)	0	
CABG + MVR	1 (10)	0	
MVR	1 (10)	0	
AVR	0	1 (10)	
AVR + MVR	0	1 (10)	
**Hypertension**	9 (90)	7 (70)	0.582
**Diabetes mellitus**	2 (20)	6 (60)	0.170
**Lactate (mmol/L)**	1.11 ± 0.42	1.34 ± 0.39	0.219
**BS (mg/dL) **	119.80 ± 54.65	159.30 ± 64.66	0.157
**Op-time (h)**	5.846 ± 0.31	5.000 ± 0.45	0.258
**Recovery time (h)**	9.50 ± 2.12	10.80 ± 2.10	0.185

Abbreviations: Dex, dexmedetomidine; CABG, coronary artery bypass grafting; BS, blood sugar.

^a^ Values are expressed as No. (%) or mean ± SD.

**Table 2.** Clinical Characteristics of Patient Pre-operation and During Surgery ^a^

Parameters	Groups		P-Value
	Control (n = 10)	Dex (n = 10)		
**SBP (mmHg)**			
Pre-op	149.50 ± 21.14	133.50 ± 19.73	0.097
30 mins after Dex	-	119.50 ± 17.71	-
Pre-pump	101.50 ± 14.73	103.00 ± 14.94	0.824
After pump off	98.50 ± 24.04	106.50 ± 12.48	0.363
Before extubation	117.00 ± 13.37	116.20 ± 16.67	0.907
After extubation	118.60 ± 13.61	123.10 ± 17.30	0.526
Post-op	123.20 ± 11.05	122.00 ± 15.73	0.846
**DBP (mmHg)**			
Pre-op	88.50 ± 18.57	75.90 ± 13.60	0.101
30 mins after Dex	-	67.70 ± 14.01	-
Pre-pump	61.20 ± 11.25	60.70 ± 9.84	0.917
After pump off	59.20 ± 13.85	58.20 ± 11.55	0.863
Before extubation	69.70 ± 11.63	65.80 ± 11.14	0.454
After extubation	72.20 ± 9.60	75.20 ± 10.92	0.522
Post-op	72.50 ± 8.85	75.10 ± 10.50	0.557
**HR (bpm)**			
Pre-op	75.20 ± 17.52	71.00 ± 12.91	0.549
30 mins after Dex	-	74.40 ± 12.36	-
Pre-pump	78.70 ± 16.64	72.50 ± 13.46	0.372
After pump off	83.10 ± 15.59	76.44 ± 10.38	0.295
Before extubation	88.50 ± 5.19	80.30 ± 11.00	0.047
After extubation	88.00 ± 10.19	84.80 ± 14.45	0.574
Post-op	88.80 ± 7.51	86.00 ± 16.27	0.627
**SPO**_**2**_ ** (%)**			
Pre-op	96.80 ± 2.62	97.40 ± 1.43	0.533
30 mins after Dex	-	98.00 ± 0.94	-
Pre-pump	97.50 ± 1.18	97.90 ± 1.29	0.478
After pump off	97.90 ± 1.60	97.50 ± 0.97	0.507
Before extubation	97.90 ± 1.29	97.80 ± 1.32	0.866
After extubation	97.70 ± 1.70	97.00 ± 2.00	0.410
Post-op	98.20 ± 1.14	96.40 ± 2.63	0.070
**PaCO**_**2**_ ** (mmHg)**			
Pre-op	38.50 ± 6.87	36.90 ± 6.21	0.591
30 mins after Dex	-	34.70 ± 4.06	-
Pre-pump	33.80 ± 7.16	36.00 ± 5.08	0.438
After pump off	36.70 ± 5.72	35.70 ± 4.55	0.670
Before extubation	37.60 ± 2.17	37.10 ± 4.36	0.750
After extubation	43.60 ± 7.57	38.90 ± 3.84	0.097
Post-op	39.80 ± 3.36	40.60 ± 4.95	0.677
**Lactate (mmol/L)**			
Before induction	1.13 ± 0.58	1.43 ± 0.63	0.301
Pre-pump	1.74 ± 1.20	1.53 ± 0.66	0.650
Pump1	2.28 ± 2.06	1.87 ± 0.90	0.576
Pump2	2.59 ± 2.33	2.12 ± 0.87	0.561
Pump3	3.78 ± 3.45	1.92 ± 0.91	0.278
Pump off	3.40 ± 2.42	2.25 ± 0.93	0.177
After closing sternum	2.27 ± 1.22	2.18 ± 1.06	0.863
24 h after-op	2.38 ± 1.58	1.91 ± 0.67	0.426
**BS (mg/dL)**			
Intra-op	153.40 ± 46.95	166.30 ± 61.51	0.605
24 h after-op	168.40 ± 68.56	177.50 ± 38.29	0.718

Abbreviations: Dex, dexmedetomidine; SBP, systolic blood pressure; DBP, diastolic blood pressure; HR, heart rate; SPO_2_, blood oxygen saturation; PaCO_2_, partial pressure of carbon dioxide; BS, blood sugar.

^a^ Values are expressed as mean ± SD.

### 4.2. Main Efficacy Measures

In the Dex group, the percentage of patients experiencing adverse effects was 0% for variables including hypertension, bradycardia, tachycardia, and hypoxia, except for one patient who exhibited hypotension (10%). In contrast, the control group met the primary endpoint, with a higher percentage of participants exhibiting adverse effects compared to the Dex group: Thirty percent experienced hypotension, 10% experienced bradycardia (also hypertension), and 20% experienced tachycardia (including one patient with reduced systolic BP after pump-off) (Table 3). Overall, 4 participants (40%) in the control group experienced one or more adverse effects, compared to only 10% (one patient) in the Dex group (Table 3). However, there was no statistically significant difference between the proportion of complications in the two groups (P = 0.303). Additionally, all events were mild in both groups.

**Table 3.** Adverse Effects of Dexmedetomidine During Pre-pump on and After Pump-off in Cardiac Surgery ^a^

Characters	Hypotension	Hypertension	Bradycardia	Tachycardia	Hypoxia	Total
**Control (n = 10)**	3 (30)	0	1 (10)	2 (20)	0	4 (40)
** Dex (n = 10)**	1 (10)	0	0	0	0	1 (10)
**P-value**	0.582	-	1	0.474	-	0.303

Abbreviation: Dex, dexmedetomidine.

^a^ Values are expressed as No (%).

**Figure 2.**
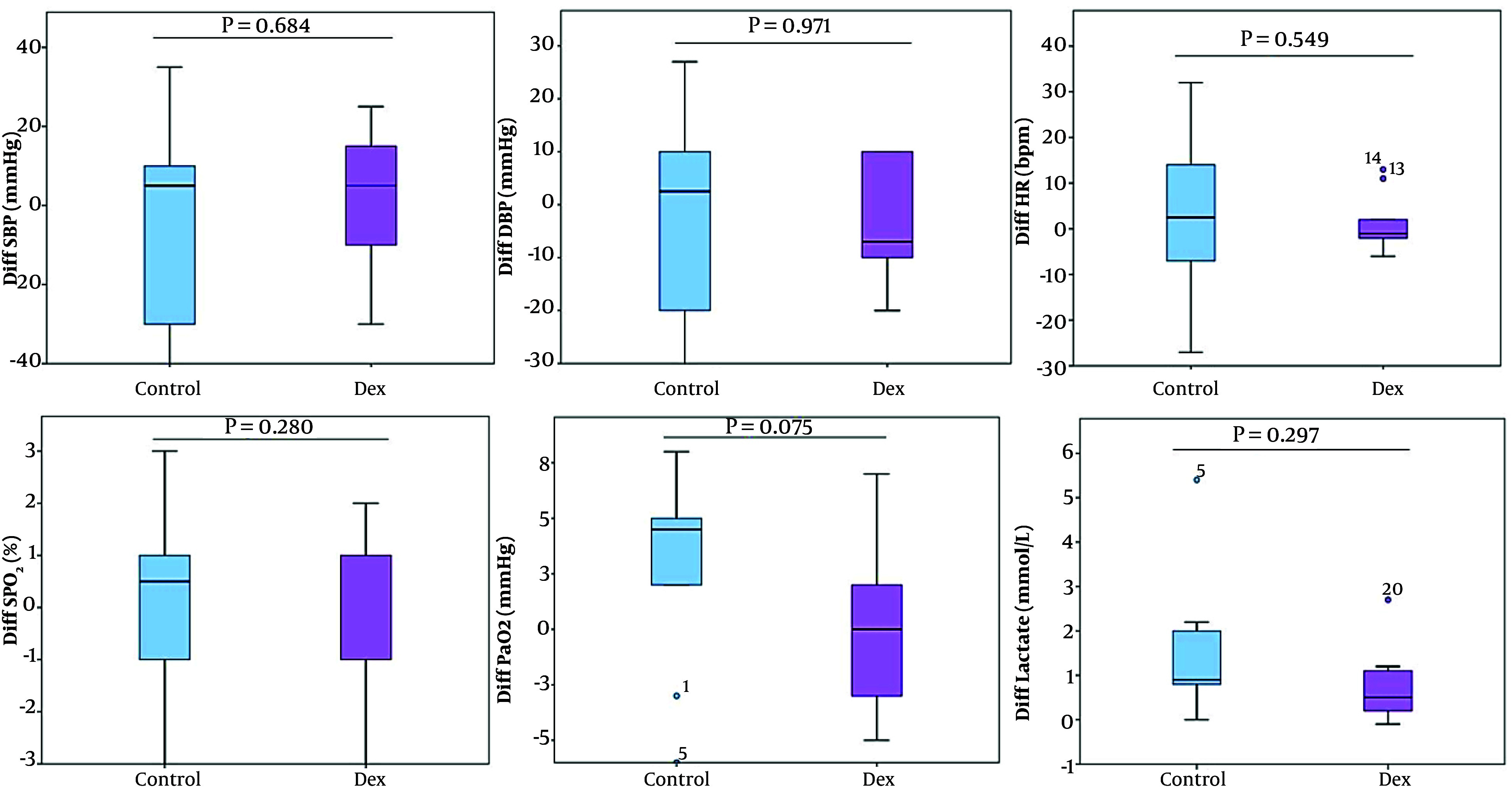
Difference before pump-on and after pump-off and variation in variables between the control and dexmedetomidine (Dex) groups

No statistically significant changes were observed in systolic blood pressure (SBP), diastolic blood pressure (DBP), HR, SpO_2_, and PaCO_2_ between the pre-pump-on and post-pump-off phases in either group. Moreover, there were no differences within the groups (Table 4 and Figure 2). Additionally, Dex demonstrated greater stability and resulted in lower changes in hemodynamic and respiratory parameters (Figure 2). 

**Table 4.** Results for the Dexmedetomidine Effects on Hemodynamic and Respiratory Stability and Laboratory Information ^a^

Characters and Groups	Pre-pump on	After Pump-off	P-Value ^b^	Changes ^c^ Median (Interquartile Range)	P-Value ^d^
**SBP (mmHg)**					0.675
Control	101.50 ± 14.73	98.50 ± 24.04	0.720	5 (-30, 10)	
Dex	103.00 ± 14.94	106.50 ± 12.48	0.442	5 (-10, 15)	
**DBP (mmHg)**					0.939
Control	61.20 ± 11.25	59.20 ± 13.86	0.766	2.5 (-20, 10)	
Dex	60.70 ± 9.84	58.20 ± 11.56	0.602	-7 (-10, 10)	
**HR (bpm)**					0.513
Control	78.70 ± 16.64	83.10 ± 15.59	0.358	2.5 (-7, 14)	
Dex	75.00 ± 11.55	76.44 ± 10.38	0.952	-1 (-2, 2)	
**SPO**_**2**_ ** (%)**					0.258
Control	97.50 ± 1.18	97.90 ± 1.60	0.470	0.5 (-1, 1)	
Dex	97.90 ± 1.29	97.50 ± 0.97	0.417	-1 (-1, 1)	
**PaCO**_**2**_ ** (mmHg)**					0.068
Control	33.80 ± 7.16	36.70 ± 5.72	0.092	4.5 (2, 5)	
Dex	36.00 ± 5.08	35.70 ± 4.55	0.610	0 (-3, 2)	
**Lactate (mmol/L)**					0.297
Control	1.74 ± 1.20	3.40 ± 2.42	0.012	0.9 (0.8, 2)	
Dex	1.53 ± 0.66	2.25 ± 0.93	0.017	0.5 (0.2, 1.1)	
**BS (mg/dL)**					0.123
Control	119.80 ± 54.65	168.40 ± 68.56	0.009	41 (28, 81)	
Dex	159.30 ± 64.66	177.50 ± 38.29	0.093	13.5 (3, 53)	

Abbreviations: SBP, systolic blood pressure; DBP, diastolic blood pressure; HR, heart rate; SPO_2_, blood oxygen saturation; PaCO_2_, partial pressure of carbon dioxide; BS, blood sugar; Dex, dexmedetomidine.

^a^ Values are expressed as mean ± SD.

^b^ Wilcoxon signed ranks test.

^c^ Changes: (After pump-off) - (pre-pump on).

^d^ Mann-Whitney test.

**Figure 3.**
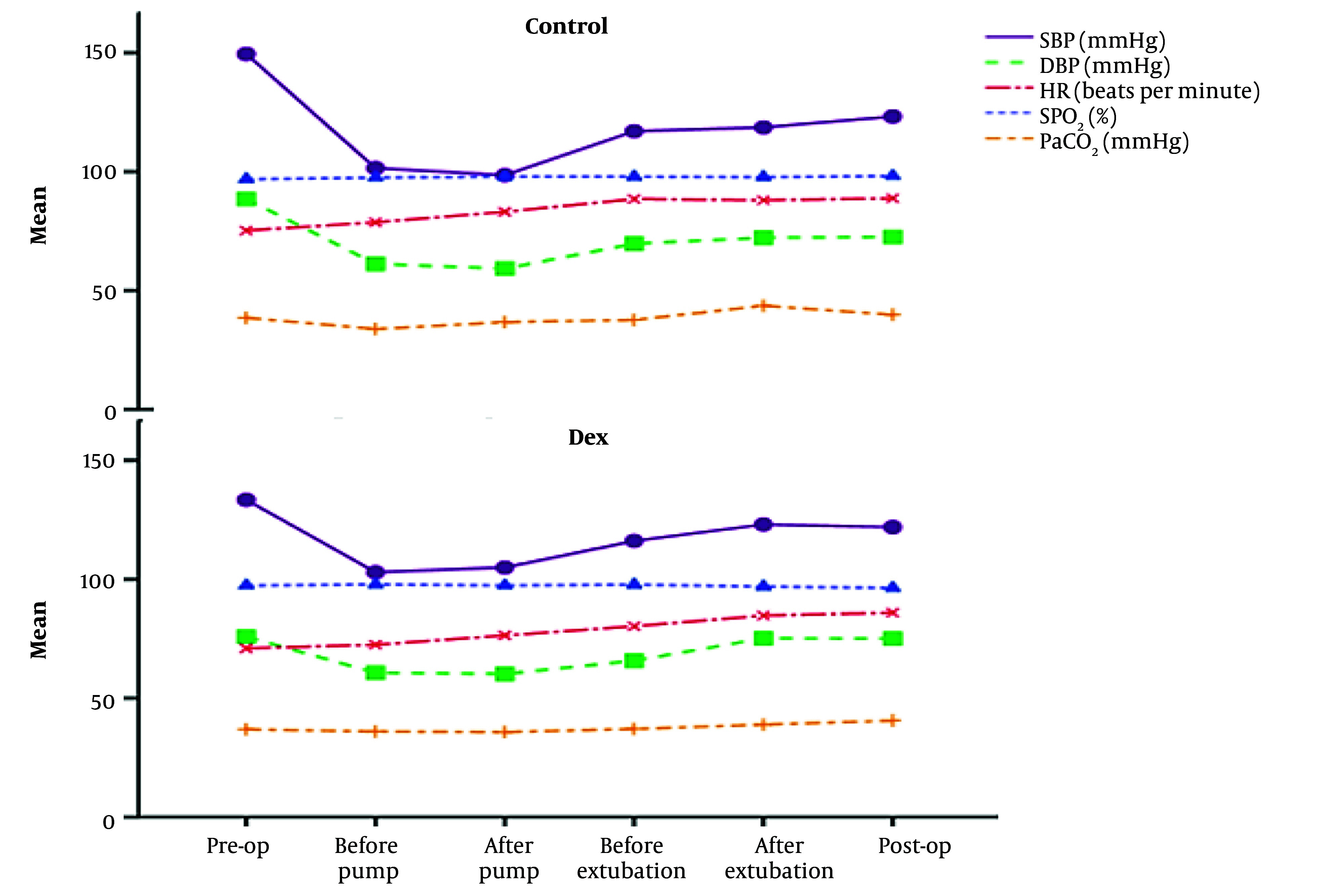
Level of variables in the control and dexmedetomidine (Dex) groups during pre-operation and post-operation time

Additionally, the hemodynamic stability was higher in the Dex group during the pre-operation and post-operation periods (Figure 3 and Appendix 2 in Supplementary File). Also, the pre-operation SBP was significantly reduced from 133.5 mmHg to 122 (Dex group, P < 0.001), and significant reduction was observed from 149.5 mmHg to 123.2 (control group, P < 0.001) by the post of operation. The control (P = 0.001) and Dex (P = 0.003) groups significantly reduced DBP between the pre-operation and post-operation period. Furthermore, Dex significantly increased HR (P = 0.004) within the group (Figure 3 and Appendix 2 in Supplementary File). These findings indicated that Dex was not only safe and without adverse effects, but also improved hemodynamic parameters and cardiac function during open-heart surgery.

### 4.3. Other Efficacy Measures

Lactate levels increased in both groups; however, Dex slightly increased lactate levels compared to the control group. In the Dex group, lactate levels were 1.53 ± 0.66 before pump-on and 2.25 ± 0.93 after pump-off (P = 0.017), while in the control group, levels were 1.74 ± 1.20 and 3.40 ± 2.42, respectively (P = 0.012) (Table 4 and Figure 2). Additionally, the increase in lactate levels was higher in the control group during and after surgery compared to the Dex group, with no statistically significant difference between the groups (Table 2 and Appendix 3 in Supplementary File).

Furthermore, there were statistically significant differences in BS levels post-operation, with the control group showing an increase from 119.80 ± 54.65 to 168.40 ± 68.56 mg/dL, compared to the Dex group, which increased from 159.30 ± 64.66 to 177.50 ± 38.29 mg/dL (Table 2 and Table 4). Dex did not significantly change BS levels (P = 0.122), while the control group showed a significant increase from baseline (P = 0.006) (Appendix 4 in Supplementary File).

The mean recovery time was similar between the control and Dex groups, at 9.50 ± 2.12 hours and 10.80 ± 2.10 hours, respectively (P = 0.185) (Table 1). 

### 4.4. Safety

The Dex failed to exhibit any adverse events during or after cardiac operations in terms of hemodynamic parameters, respiratory stability, and laboratory data, as shown in Table 3 and Table 4. 

## 5. Discussion

In our study, the administration of Dex in open heart surgery patients did not result in a significant majority of adverse reactions (such as respiratory depression, hypotension, or hypoxia) compared to the control group, indicating the effectiveness and safety of Dex. Additionally, all secondary endpoints supported the safety and efficacy of Dex, with no significant differences between groups. However, the results of this study showed that Dex, as an adjunct to the anesthesia protocol in patients undergoing open heart surgery, had corresponding adverse effects, without significant changes before and during pumping during anesthesia. In previous studies, Dex infusion in cardiac surgery was reported to cause hypotension, bradycardia, hemodynamic dysfunction, and arrhythmia (25). While our study demonstrated the safety of Dex infusion, with no patients experiencing hypotension, hypertension, bradycardia, tachycardia, or hypoxia, there was no significant difference observed in BP, SpO_2_, PaCO_2_, hemodynamic characteristics, and levels of lactate dehydrogenase (LDH) and BS between the groups. Moreover, patients in the Dex group exhibited greater hemodynamic and biological (lactate and BS) stability compared to the control group during Dex infusion and after cardiac surgery. Our findings align with those of Leung et al.'s cohort study, which reported the safety of high-dose Dex infusion after cardiac surgery (26). Furthermore, Dex played a plausible and safe role in managing hemodynamic (hypotension, hypertension, bradycardia, tachycardia) and respiratory (hypoxia or respiratory depression) side effects in non-ICU patients receiving anesthesia (23).

Interestingly, no incidence of bradycardia or tachycardia was observed in the Dex group, compared to the control group, which experienced rates of 10% (1/10 patients) and 20% (2/10 patients), respectively. The frequency of hypotension was higher in the control group than in the Dex group, at 30% (3/10 patients) and 10% (1/10 patients), respectively. Since no patient in either group suffered from hypoxia or required intubation, this may indicate that respiratory depression did not occur during the infusion of Dex in patients undergoing cardiac surgery. These observations suggest the safety and absence of adverse events associated with Dex regarding the infusion protocol and the dose used in this study. Therefore, it appeared that all hemodynamic and respiratory parameters remained stable during Dex infusion, and there was no significant difference in recovery time between groups. This evidence is consistent with previous studies showing that Dex may be safe, with no adverse effects on BP, arrhythmias (bradycardia, tachycardia), and HR in cardiac or other surgical patients (27-32). However, some hemodynamic effects observed in the present study may be explained by the sympatholytic and vasoconstrictive roles of Dex as an α2-adrenoceptor agonist (29). Therefore, Dex may have significant potential to prevent hemodynamic side effects during cardiac surgery.

Although our data failed to show a significant difference between the groups in lactate levels, the change from before pump to after pump was greater in the control group than in the Dex group. However, the pattern of lactate levels in both groups was incremental (hyperlactatemia without metabolic acidosis), which was statistically significant between the pre-pump and post-pump phases within each group. Moreover, Dex increased lactate levels with lower intensity compared to the control, which may suggest that Dex enhances lactate clearance through its intrinsic effect as a selective α-2-adrenergic agonist and its sympatholytic role (33). There is evidence indicating that inflammation, diabetes, surgery, hypoxia, and cancer can lead to increased lactate levels (34-36). Conversely, previous studies have reported that Dex suppresses inflammatory stress responses, sympathetic nervous activation, and hypoxia, which are stimulated by cardiac surgery (5, 6, 29). Moreover, Dex has been shown to decrease lactate levels through modulation of adrenergic activity in sheep with septic shock (33). Therefore, Dex may improve circulation in cardiac surgery by reducing lactate levels in conjunction with hemodynamic stability, as observed in this study.

### 5.1. Conclusions

Given that no adverse effects were observed with this administration protocol of Dex, it appears to help reduce hemodynamic variability and improve circulation without causing hypotension, hypoxia, bradycardia, or other significant effects. There was no significant reduction in either systolic or diastolic BPs with Dex infusion, and no changes in HR, hypoxia, or respiratory parameters during Dex administration. Importantly, Dex infusion was found to be safe, as supported by hemodynamic stability, laboratory, and respiratory data. Furthermore, Dex slightly increased lactate levels. These results provide valuable insights into the use of Dex as a safe anesthetic agent in patients undergoing cardiac surgery. However, further clinical studies with larger populations and additional evaluations, such as inflammatory mediators, are required to validate these results and provide more reliable evidence.

### 5.2. Limitations

The present study had several limitations. The small sample size may have affected the study's power and results. Changes in some variables were not investigated in a time-dependent manner, particularly during surgery. Additionally, patients' inflammatory mediators, ICU or hospital stay durations, and laboratory markers were not measured. Another limitation was the inability to include the timeline of adverse events, required interventions, and their clinical impact in our study due to operating room constraints.

aapm-15-1-157117-s001.pdf

## Data Availability

The dataset presented in the study is available on request from the corresponding author during submission or after its publication. The data are not publicly available due to privacy.

## Clinical Trial Registration Code:

IRCT20240624062233N1.
