# Supplementary material for: Double-Blind, Randomized, Controlled Pilot Trial to Specify Collateral Effect and Safety of Perioperative Dexmedetomidine in Patients Undergoing Open Heart Surgery
Source: Anesth Pain Med. 2025 Feb 22;15(1):e157117. doi: 10.5812/aapm-157117 (PMC12125658; doi:10.5812/aapm-157117)
Supplement: aapm-15-1-157117-s001.pdf [file aapm-15-1-157117-s001.pdf]

Appendix 1. Range of hemodynamic and respiratory criteria as adverse events.

Parameter	Measured value	Adverse event name	Ref.
**SBP**	< 80 mmHg or ≥ 30% decrease from baseline	Hypotension	(23)
	> 180 mmHg or ≥ 30% increase from baseline	Hypertension	
**DBP**	< 50 mmHg	Hypotension	(23)
	> 100 mmHg	Hypertension	
**Heart rate**	< 40 bpm or ≥ 30% decrease from baseline	Bradycardia	(23)
	> 120 bpm or ≥ 30% increase from baseline	Tachycardia	
**SpO_2**	< 90% or > 10% decrease from baseline	Hypoxia	(23)

bpm, beats per minute; DBP, diastolic blood pressure; SBP, systolic blood pressure; SpO_2, oxygen saturation.

Appendix 2. The change of hemodynamic and respiratory parameters before and during dexmedetomidine infusion and postoperative cardiac surgery.

Characters	Group	Pre-Op Mean ± SD	Pre-Pump Mean ± SD	After Pump Off Mean ± SD	Before Extubation Mean ± SD	After Extubation Mean ± SD	Post-Op Mean ± SD	P-value*
**SBP (mmHg)**	Control	149.5 ± 21.1	101.5± 14.7	98.50 ± 24.04	117.00 ±13.4	118.60 ±13.6	123.2 ±11.1	<0.001
	Dex	133.5 ± 19.7	103.0 ±14.9	106.50 ± 12.48	116.20 ±16.7	123.10 ±17.3	122.0 ±15.7	<0.001
**DBP (mmHg)**	Control	88.50 ± 18.5	61.20 ±11.2	59.20 ± 13.86	69.70 ± 11.6	72.20 ± 9.60	72.50 ± 8.85	0.001
	Dex	75.90 ± 13.6	60.70 ±9.84	58.20 ± 11.56	65.80 ± 11.1	75.20 ±10.92	75.10 ±10.5	0.003
**HR (bpm)**	Control	75.20 ± 17.5	78.70 ±16.6	83.10 ± 15.59	88.50 ± 5.19	88.00 ±10.19	88.80 ± 7.51	0.357
	Dex	73.11 ± 11.7	75.00 ±11.5	76.44 ± 10.38	79.22 ±11.09	83.33 ±14.52	82.78 ±13.5	0.004
**SPO_2 (%)**	Control	96.80 ± 2.62	97.50 ±1.18	97.90 ± 1.60	97.90 ± 1.29	97.70 ± 1.70	98.20 ± 1.14	0.445
	Dex	97.40 ± 1.43	97.90 ±1.29	97.50 ± 0.97	97.80 ± 1.32	97.00 ± 2.00	96.40 ± 2.63	0.480
**$PaCO_2$ (mmHg)**	Control	38.50 ± 6.87	33.80 ±7.16	36.70 ± 5.72	37.60 ± 2.17	43.60 ± 7.58	39.80 ± 3.36	0.007
	Dex	36.90 ± 6.21	36.00 ±5.08	35.70 ± 4.55	37.10 ± 4.36	38.90 ± 3.84	40.60 ± 4.95	0.191

*: within-group (Friedman Test).

Appendix 3. Lactate level before and during dexmedetomidine infusion.

Lactate (mmol/L)	Groups			
	Control		Dex	
	Mean ± SD	Median (IQR)	Mean ± SD	Median (IQR)
Pre-Op	1.00 ± 0.26	1.00 (0.72, 1.20)	1.38 ± 0.42	1.30 (1.13, 1.48)
Before Induction	0.98 ± 0.35	0.90 (0.63, 1.30)	1.45 ± 0.71	1.30 (1.15, 1.45)
Pre-Pump	1.5 ± 1.07	1.15 (0.93, 1.55)	1.63 ± 0.65	1.35 (1.20, 2.13)
Pump1	2.19 ± 2.19	1.25 (1.20, 2.13)	1.93 ± 1.00	1.50 (1.30, 2.48)
Pump2	2.49 ± 2.47	1.45 (1.30, 2.43)	2.09 ± 0.97	1.75 (1.45, 2.38)
Pump Off	3.19 ± 2.66	2.10 (1.85, 3.57)	2.10 ± 0.93	1.70 (1.42, 2.60)
After Closing Sternum	2.06 ± 0.64	2.05 (1.53, 2.30)	2.24 ± 1.11	2.00 (1.33, 2.78)
Post-Op	2.36 ± 1.68	1.50 (1.22, 4.28)	1.75 ± 0.50	1.55 (1.40, 2.22)
**P-value***		**<0.001**		**0.012**

IQR: interquartile range, *: within-group (Friedman Test).

Appendix 4. Blood sugar level before and during dexmedetomidine infusion.

Character		Per-Op Median (IQR)	Intra-Op Median (IQR)	Post-Op Median (IQR)	P-value*
**Blood sugar (BS) (mg/dL)**	Control	103 (86.75,126.75)	131 (115,190)	140.5 (120.5, 208.25)	0.006
	Dex	160 (116.75,186.25)	162.5 (124.25, 209)	172.5 (149, 208)	0.122

IQR: interquartile range, *: within-group (Friedman Test).
